# *PHACTR1*, a coronary artery disease risk gene, mediates endothelial dysfunction

**DOI:** 10.3389/fimmu.2022.958677

**Published:** 2022-08-25

**Authors:** Xiaoxuan Ma, Meiming Su, Qingze He, Zhidan Zhang, Fanshun Zhang, Zhenghong Liu, Lu Sun, Jianping Weng, Suowen Xu

**Affiliations:** Department of Endocrinology, Institute of Endocrine and Metabolic Diseases, The First Affiliated Hospital of USTC, Division of Life Sciences and Medicine, Clinical Research Hospital of Chinese Academy of Sciences (Hefei), University of Science and Technology of China, Hefei, China

**Keywords:** endothelial dysfunction, inflammation, PHACTR1, cardiovascular disease, RNA sequencing

## Abstract

Genome-wide association studies (GWAS) have recently identified phosphatase and actin regulator-1 (PHACTR1) as a critical risk gene associated with polyvascular diseases. However, it remains largely unclear how PHACTR1 is involved in endothelial dysfunction. Here, by mining published datasets of human stable and vulnerable/ruptured plaque tissues, we observed upregulated expression of *PHACTR1* in vulnerable/ruptured plaques. Congruent with these data, we demonstrated increased *Phactr1* gene expression in aortic endothelium from ApoE^-/-^ mice fed a western type diet compared with that in normal C57BL/6J mice. Relevantly, *PHACTR1* gene expression was upregulated by pro-inflammatory and pro-atherogenic stimuli, including TNF-α, IL-1β and oxidized LDL (oxLDL). By employing next-generation RNA sequencing, we demonstrate that PHACTR1 overexpression disrupts pathways associated with endothelial homeostasis. Cell biological studies unravel that PHACTR1 mediates endothelial inflammation and monocyte adhesion by activating NF-κB dependent intercellular adhesion molecule 1 (ICAM1) and vascular cell adhesion molecule 1 (VCAM1) expression. In addition, overexpression of PHACTR1 also reduces the generation of nitric oxide (NO) by inhibiting Akt/eNOS activation. In-house compound screening of vasoprotective drugs identifies several drugs, including lipid-lowering statins, decreases PHACTR1 gene expression. However, PHACTR1 gene expression was not affected by another lipid-lowering drug-fenofibrate. We also performed a proteomic study to reveal PHACTR1 interacting proteins and validated that PHACTR1 can interact with heat shock protein A8 (HSPA8) which was reported to be associated with coronary artery disease and eNOS degradation. Further studies are warranted to confirm the precise mechanism of PHACTR1 in driving endothelial dysfunction. In conclusion, by using systems biology approach and molecular validation, we disclose the deleterious effects of PHACTR1 on endothelial function by inducing endothelial inflammation and reducing NO production, highlighting the potential to prevent endothelial dysfunction and atherosclerosis by targeting PHACTR1 expression. The precise role of endothelial cell PHACTR1 in polyvascular diseases remains to be validated in diseased conditions.

## Introduction

Coronary artery disease (CAD) has been a major cause of increasing morbidity and mortality of global human population (1). Genome-wide association studies (GWAS) have identified phosphatase and actin regulator-1 (*PHACTR1)* as a risk gene associated with coronary artery disease (CAD) recently (2). However, it remains largely unclear how these GWAS-associated genes affect the development of CAD. Endothelial dysfunction plays an important role in the initiation and progression of CAD (3, 4). Vascular endothelial dysfunction consists of chronic inflammation, impaired endothelium-dependent vasodilation, oxidative stress, leukocyte adhesion and hyperpermeability, and endothelial-to-mesenchymal transition (5).

ECs are the central and active players in the immune system and vasculature (6). Although ECs are not professional immune cells, they essentially execute immune functions by sensing and responding to inflammatory cytokines, viral and bacterial infection (7). Upon inflammation, ECs recruit immune cells, such as neutrophils and monocytes and direct the extravasation of leukocytes at the inflammatory site through inducing the adhesion molecules like vascular cell adhesion molecule 1 (VCAM1) or intercellular adhesion molecule 1 (ICAM1) and E-selectin (SELE) expression (8). We have previously demonstrated that GWAS-identified CAD risk gene JCAD/KIAA1462 regulates leukocyte adhesion and atherosclerosis in mice *via* regulating endothelial inflammation (9). It remains elusive whether other GWAS-CAD genes will also affect endothelial inflammation and dysfunction.

Emerging studies have implicated phosphatase and actin regulator 1 (*PHACTR1*) as another important GWAS-identified CAD risk gene associated with polyvascular disease, including CAD, migraine, hypertension, fibromuscular dysplasia and cervical artery dissection (10, 11). *PHACTR1* encodes a protein that binds to actin and protein phosphatase 1 (PP1), which plays a role in endothelial survival and tube formation (12, 13). Recently, a comprehensive integrative genomics analysis has reported the top 25 prioritized candidate risk genes for CAD. *PHACTR1* ranked the second among the list of 25 prioritized candidate causal genes (14). PHACTR1 is essential for macrophages efferocytosis capacity and facilitates M2 macrophage polarization to inhibit atherosclerosis in mice (15, 16). In addition, PHACTR1 regulates the proliferation, apoptosis, migration and tube formation of ECs (12, 13, 17). PHACTR1 also promotes vascular calcification in vascular smooth muscle cells (18). In ECs, PHACTR1 is inextricably linked with endothelial inflammation and endothelial dysfunction; however, the precise role of PHACTR1 in regulating endothelial function remain controversial. In one study, downregulation of PHACTR1 trigged pro-inflammatory and pro-atherogenic factors, including CD36, cadherin-13, PAR-1 and thrombin (19). In another study, the authors have showed that PHACTR1 drived oxidative stress and inflammation through interaction with NF-κB/p65 in human coronary artery endothelial cells (HCAECs) (20). However, the effect and molecular mechanism whereby PHACTR1 modulates endothelial function and homeostasis remain largely unknown.

Therefore, the purpose of the present study is to evaluate the effect and mechanism of PHACTR1 overexpression on endothelial dysfunction by focusing on inflammation and nitric oxide (NO) production. By utilizing unbiased RNA-sequencing and subsequent molecular characterizations, we demonstrate that PHACTR1 overexpression mediates endothelial inflammation and impairs endothelial NO production to render endothelial dysfunctional.

## Materials and methods

### Chemicals and reagents

TNF-α (#300-01A, PEPROTECH, Rocky Hill, USA); IL-1β (#200-01B; PEPROTECH, Rocky Hill, USA); oxLDL (#H7980, Solarbio, Beijing, China); DMSO (#A100231, Sangon Biotech, Shanghai, China); Atorvastatin (#222412-82-0, Cayman Biochem, Ann Arbor, Michigan, USA); Simvastatin (#79902-63-9, TargetMol, Shanghai, China); Rosuvastatin (#287714-41-4, TargetMol, Shanghai, China); Fenofibrate (#49562-28-9,TargetMol, Shanghai, China); Empagliflozin (#864070-44-0,TargetMol, Shanghai, China); Troglitazone (#97322-87-7, MCE, Shanghai, China); Metformin (#1115-70-4,TargetMol, Shanghai, China); Cilostazol (#73963-72-1,TargetMol, Shanghai, China); Fluvoxamine (#61718-82-9,TargetMol, Shanghai, China); Riociguat (#625115-55-1,TargetMol, Shanghai, China); Sildenafil citrate (#171599-83-0,TargetMol, Shanghai, China).

### Mice and diet

C57BL/6J and ApoE^-/-^ mice were purchased from Gempharmatech (Nanjing, China). ApoE^-/-^ mice were fed with 1.25% High Cholesterol Diet (#D12108C, RESEARCH DIETS, NJ, USA) and C57BL/6J mice were fed with chow diet for 6 weeks. After then, mice were sacrificed and endothelium-enriched intimal RNA was collected. The Institutional Animal Care and Use Committee of University of Science and Technology of China (USTC) approved all animal care procedures. All experiments were performed in accordance with the relevant regulations by USTC.

### Oil Red O staining

After sacrifice, mouse hearts were fixed with 4% PFA (G1101, Servicebio, Wuhan, China) and then dehydrated with 30% sucrose solution. Heart tissues were embedded by OCT for whole night, and then frozen sections of the aortic sinus were performed with a thickness of 8 µm. Frozen sections were rinsed with 10% isopropyl alcohol (#A507048, Sangon Biotech, Shanghai, China) after rewarming. The aortic sinus was stained with 0.3% Oil Red O solution (#G1260, Solarbio, Wuhan, China) for 1 min, rinsed with 10% isopropyl alcohol for 10 s, and rinsed with PBS for several minutes. 10% Fast Green solution (#G1661, Solarbio, Wuhan, China) was applied for counterstaining for 10 s, and rinse with PBS for 1 min. Finally, slides were sealed with glycerin gelatin mounting media. The images were taken using Pannoramic MIDI (3D HISTECH, Hungary).

### HUVECs culture

Human umbilical vein endothelial cells (HUVECs) were isolated from the umbilical cord of normal pregnant women as previously described (21). The clinical scheme of umbilical cord collection in this experiment was approved by the institutional review committee of the First Affiliated Hospital of University of Science and Technology of China (scheme No.: 2020-ky013). HUVEC cells within 3-9 generations from three to four different donors were used in this study. The culture temperature of these cells was 37 °C, and the gas environment contained 5%CO_2_. ECM medium was used to culture these cells, which consists of 1×endothelial cell growth supplement (ScienCell, Carlsbad, CA), 5% FBS and 1×penicillin/streptomycin antibiotic.

### RNA extraction, reverse transcription and RT-qPCR

RNeasy kits (YiShan Biotech, Shanghai, China) were used to extract total RNA from cultured HUVECs. In addition, Reverse Transcription Kits (Takara, Dalian, China) or HiScript III RT SuperMix (Vazyme, Nanjing, China) were used to convert the total RNA of HUVECs into complementary cDNA. After the reverse transcription process, ChamQ SYBR qPCR Master Mix (Vazyme, Nanjing, China) was used in quantitative real-time PCR assay. Meanwhile, Roche LC96 real-time PCR detection system was used for detection, and GAPDH was used as loading control for relative mRNA quantification. **Supplementary Table 1** lists the sequences of primers used.

### Western blot

Whole-cell lysates were extracted from HUVECs with sample buffer and boiled at 95 °C for 10 min. Samples were separated by SDS-PAGE, and then transferred to nitrocellulose membrane (Pall, New York, USA). The nitrocellulose membrane was incubated with blocking buffer at room temperature for 1 hour. After discarding the blocking buffer, the blots were incubated with primary antibodies at 4 °C overnight. The primary antibodies used were listed in **Supplementary Table 2**. 1 × Tris buffered saline with 0.1% Tween-20 (TBST) was used to wash the membrane three times for 10 minutes each time. Then, membranes were incubated with IRDye^®^ 680RD Goat anti-Mouse IgG (H + L) or IRDye^®^ 800CW Goat anti-Rabbit IgG (H + L) (1:10,000 dilution, LI-COR, Lincoln, Nebraska, USA) at room temperature for 1 hour. Finally, the Li-COR CLx infrared imaging system was used to visualize the blots.

### RNA transfection

HUVECs at sub-confluence were seeded in 12-well plate the day before transfection. The culture medium was changed into serum-free Opti-MEM medium with 350 μL per well. Dilution of transfection reagent lipofectamine2000 (lipo2000): before use, mix the lipo2000 transfection reagent gently, then take 3 μL of lipo2000 from each well. Serum free opti-MEM was used to dilute lipo2000. Dilution of siRNA (#SIGS0016696-1, si-PHACTR1#1, RIBOBIO, Guangzhou, China): Serum free opti-MEM was used to dilute control or *PHACTR1* siRNA (20 nM). Fresh complete culture medium was added 4 h after transfection. Cells were cultured for 48 h since transfection before RNA or protein was collected.

### Dual-luciferase reporter assay

HUVECs were seeded in 12-well plates. Stable-Lite Luciferase Assay System (#DD1202-01, Vazyme, Nanjing, China) was used to determine the NF-κB-luciferase activity. Ad-NF-κB-luc (Vector Biolabs, Malvern, PA, USA M.O.I. = 1) was used to transfect HUVECs for 48 h. Afterwards, HUVEC was treated with Ad-*PHACTR1* (#NM_030948, WZ Biosciences, Shandong, China) or control adenovirus for 24 h and treated with TNF-α (10 ng/ml) for 3 h. Lysis buffer was added to treated cells, and normalized luciferase activity was measured with a microplate reader (Molecular Devices, iD3).

### THP1 monocyte adhesion assay

HUVECs were seeded in 12-well plates and were treated with Ad-*PHACTR1* or control adenovirus for 24 h. Then, HUVECs were treated with TNF-α (10 ng/ml) for 6 h to activate inflammatory response. THP1 monocytic cells were seeded onto the monolayer of HUVECs at 1 × 10^5^ cells/well for an additional 30 min. After three gentle rinses with ECM medium, non-adherent THP1 monocytes were removed. A Zeiss microscope was used to image adherent monocytes, and each well was photographed. Then, the number of adherent monocytes was obtained by counting cells in each well, and the value was calculated and presented.

### The NO production assay

DAF-FM DA dye (#S0019, Beyotime, Shanghai, China) was diluted with the DAF-FM DA diluent provided with the kit at a dilution of 1:1, 000 to reach a final concentration of 5 μM. After digestion, the cells were counted, centrifuged, and resuspended with diluted DAF-FM DA. Usually, the volume of diluted DAF-FM DA is 200 μL per each well in the 12-well plate. Cells were incubated with DAF-FM DA for 20 min at 37°C. After staining, the cells were washed three times with PBS (pH7.4). Fluorescence intensity was detected by a fluorimeter (SYNERGY H1, BioTek, USA) using excitation wavelength of 495 nm and emission wavelength of 515 nm.

### Immunoprecipitation

HUVECs were treated with control adenovirus or Ad-*PHACTR1* for 24 h before stimulation of TNF-α for 6 h. Cells were lysed in IP lysis buffer (150 mM NaCl, 2.5 mM KCl, 10 mM Tris, pH 7.5, 30 mM β-glycerophosphate, 0.5% Triton X-100, 0.5% Nonidet P-40, and 50 mM NaF) supplemented with 1% protease inhibitor cocktail. 10% of the lysate was saved as input, while the remaining lysate were incubated with Anti-flag^®^ M2 affinity gel (#A2220, Sigma, Saint Louis, USA) overnight at 4°C. Beads were washed 10 times using IP lysis buffer before elution with loading buffer, followed by boiling with heated block (95°C, 10 min). SDS-PAGE gel was prepared and gel samples were cut at 1 cm from the top of the separation gel. Gel slices were sent for mass spectrometry analysis (PTM BIO, Hangzhou, China).

### RNA-seq

HUVECs were seeded in 0.1% gelatin-coated dishes the day before experiment. One day later, cells were treated with Ad-*PHACTR1* (MOI=1, WZ Biosciences) or control adenovirus for 24 h. After treatment, an RNA-Easy Mini Plus kit (QIAGEN, Germany) was used to isolate the total RNA. RNA libraries were prepared for sequencing by BGI (Beijing Genomic Institute in ShenZhen, China) as previously described (22).

### PHACTR1 interactomics study

Gel slices were treated with 50% acetonitrile for decolorization, followed by 100% acetonitrile treatment for dehydration, and subject to vacuum drying for 15 min. Trypsin was added at the final concentration of 10 ng/μL to digest the gel overnight at 37°C. The peptides were separated by an ULTRA-performance liquid phase system and then injected into an NSI ion source for ionization and analysis by Q-EXactive ™ Plus mass spectrometry. Retrieval parameter settings were as follows: The annotated database was homo_sapiens_9606_SP_20210721.FASTA (20387 sequences), and the reverse database was added to calculate the false positive rate (FDR) caused by random matching. Common contamination database was added to eliminate the influence of contaminated proteins in identification results. The number of missing tangent position is set to 2; The minimum peptide length was set to 7 amino acid residues; The maximum modification number of peptide was set to 5. FDR for protein identification and PSM identification was set to 1%.

### Quantification and statistical analysis

In this study, unless specified otherwise, means ± SD is used to represent data. Graphpad Prism software version 9.0 (Graphpad software, La Jolla, CA) is used for graphing and statistical analysis. When appropriate, students t-test or one-way analysis of variance (ANOVA) were used to test and analyze the data when appropriate. *P* value less than 0.05 is considered to be statistically significant.

## Results

### *PHACTR*1 is an important CAD risk gene that mediates endothelial dysfunction.

**Figure 1:**
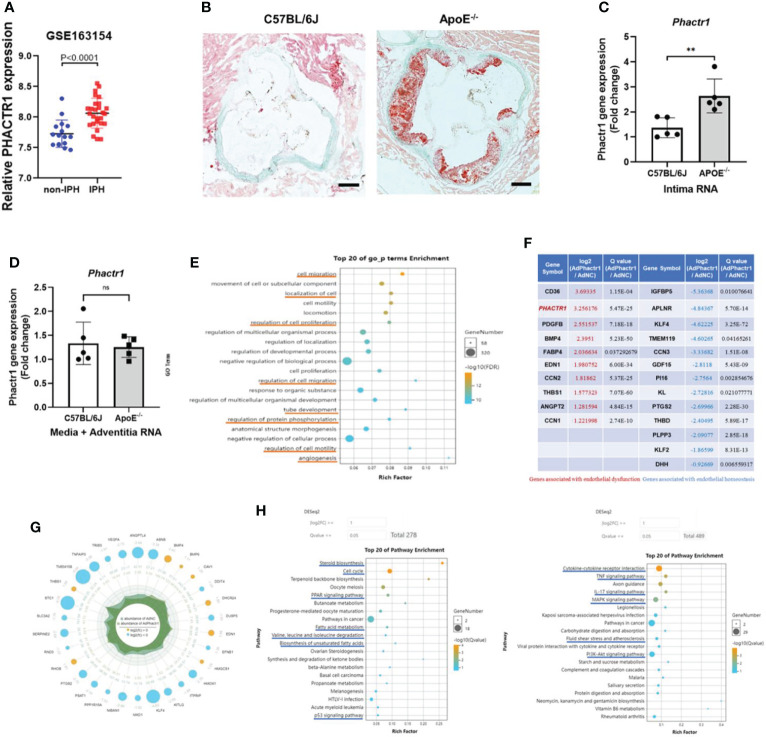
PHACTR1 is an important CAD risk gene that mediates endothelial dysfunction. **(A)** Expression of PHACTR1 mRNA levels in unstable plaques/intraplaque hemorrhage (IPH) (n = 27) and stable plaques (non-IPH, n = 16) from human plaques mined from GSE163154. **(B)** Oil Red O staining of lesions of the aortic root in male ApoE-/-mice following 6 weeks of western diet and male C57BL/6J WT mice fed with normal chow diet, n = 5. Scale bar = 200 μm. **(C)** Phactr1 gene expression in vascular intimal lysate of ApoE-/- mice compared with C57BL/6J WT mice (n = 5 mice per group), **P < 0.01. **(D)** Phactr1 gene expression in vascular media and adventitia of ApoE-/- mice compared with C57BL/6J WT mice (n = 5 mice per group) ns, non-significant. **(E)** Top 20 GO_p terms enrichment of Ad-PHACTR1-treated endothelial cells revealed by transcriptomic profiling. **(F)** Impact of PHACTR1 overexpression on selected expression of genes relevant to endothelial function dysfunction by transcriptomic profiling. **(G)** Differential genes with top 30 P value of Ad-PHACTR1-treated endothelial cells. **(H)** Top 20 KEGG upregulated or downregulated pathways enriched in Ad-PHACTR1-treated endothelial cells.

*PHACTR1* is a GWAS-identified gene associates with polyvascular diseases (11). We first mined data from dataset in GSE163154, which showed that gene expression of *PHACTR1* is significantly increased in aortic plaque tissues from patients with intraplaque hemorrhage (IPH) vs those without IPH (non-IPH) (**Figure 1A**). Moreover, by mining another GEO datasets (GSE41571) (23), we found that *PHACTR1* gene expression was upregulated in macrophage-rich regions of ruptured human atheromatous plaques (using laser micro-dissection), compared with that of stable plaques (**Supplemental Figure 1**). Meanwhile, we found that *Phactr1* gene expression was upregulated in vascular intima of ApoE^-/-^mice fed with western diet for 6 weeks compared with C57BL/6J mice (**Figures 1B, C**). However, *Phactr1* gene expression in media/adventitia from ApoE^-/-^ mice and C57BL/6J mice was similar (**Figure 1D**). To understand the potential effects of PHACTR1 on endothelial function, we performed RNA sequencing of HUVECs with *PHACTR1* overexpression. We obtained top 20 GO_p terms enrichment of Ad-*PHACTR1*-treated HUVECs. The differentially expressed genes upon *PHACTR1* overexpression relate to cell migration, localization of cells, regulation of cell proliferation and motility, tube development and angiogenesis, and regulation of protein phosphorylation (**Figure 1E**). Coincidentally, there were several studies revealing the role of *PHATCR1* on biological processes above (12, 13, 17). The analysis of differentially expressed genes showed that some genes associated with endothelial dysfunction were significantly upregulated, such as CD36, EDN1, ANGPT2 and so on. While genes maintaining endothelial homeostasis were downregulated, such as KLF4, KLF2, THBD and so on. This suggests that *PHACTR1* overexpression may aggravate endothelial dysfunction (**Figure 1F**). Similarly, among ranking top 30 minimum P value genes, bule bubbles representing for upregulated genes were associated with endothelial dysfunction, while yellow bubbles representing for downregulated genes were associated with maintaining endothelial homeostasis (**Figure 1G**). **Figure 1H** showed the top 20 KEGG pathways enriched with upregulated and downregulated differential genes, respectively. Collectively, *PHACTR1* may be an important CAD risk gene that mediates endothelial dysfunction.

### PHACTR1 mediates endothelial inflammation

**Figure 2:**
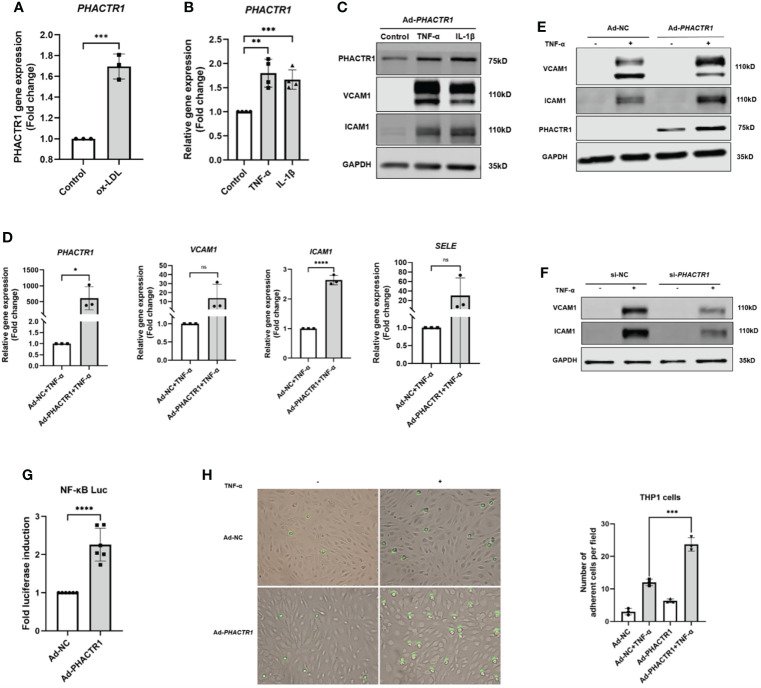
PHACTR1 mediates endothelial inflammation. **(A)** PHACTR1 mRNA level was upregulated by ox-LDL (50 μg/mL, 6 h) in HUVECs (n = 3). **(B)** PHACTR1 mRNA level was upregulated under TNF-α and IL-1β (10 ng/mL, 24 h) in HUVECs (n = 4). **(C)** PHACTR1 protein level was upregulated under TNF-α and IL-1β (10 ng/mL, 24 h) in HUVECs (n = 5). **(D, E)** PHACTR1 overexpression enhanced VCAM1, ICAM1 gene and protein expression under TNF-α stimulation (10 ng/mL, 6 h) in HUVECs (n = 3 or 6). **(F)** PHACTR1 silencing suppressed VACM1 and ICAM1 protein expression under TNF-α stimulation (10 ng/mL, 6 h) in HUVECs (n = 3). **(G)** PHACTR1 overexpression activated NF-κB luciferase activity (n = 6). **(H)** PHACTR1 overexpression aggravated the adhesion of THP1 cells under TNF-α stimulation (10 ng/mL, 6 h) in HUVECs (n = 3). ns, non-significant, *P < 0.05, **P < 0.01, ***P < 0.001, ***P < 0.0001 ****, P < 0.0001.

Oxidized low-density lipoprotein (ox-LDL) is considered as the main contributing factor to endothelial damage and atherosclerosis (24). HUVECs was treated with 50 μg/mL for 6 h, and then we found that *PHACTR1* gene expression was upregulated by ox-LDL (**Figure 2A**). This piece of data agreed with a previous report by Reschen *et al.* who found that an intermediate length transcript of *PHACTR1* was upregulated by ox-LDL (25). Moreover, tumor necrosis factor (TNF-α) and interleukin-1β (IL-1β), two additional inducers of inflammatory response by upregulated expression of endothelial adhesion molecules (26–28). These adhesion molecules (e.g., VCAM1 and ICAM1) regulate endothelial barrier function. These molecules also mediate leukocyte transendothelial migration and endothelial permeability during inflammation (29, 30). When HUVECs were treated with the TNF-α or IL-1β, we observed that both *PHACTR1* mRNA and PHACTR1 protein expression were upregulated, suggesting that PHACTR1 responds to inflammatory stimulation (**Figures 2B, C**). Then, we examined the expression of adhesion molecules (VCAM1 and ICAM1) upon overexpressing or silencing *PHACTR1* in HUVECs. The results showed that the protein expression of inflammatory markers VCAM1 and ICAM1 were upregulated through overexpression of *PHACTR1* (**Figures 2D, E**). Meanwhile, knocking down *PHACTR1* downregulated protein expression of VCAM1 and ICAM1 (**Supplemental Figure 2** and **Figure 2F**). The above results demonstrated that PHACTR1 mediates endothelial inflammation. Next, we wanted to validate whether endothelial inflammation induced by *PHACTR1* is triggered by typical pro-inflammatory NF-κB pathway. Through assessing the activation of NF-κB by luciferase reporter assay, we observed that the overexpression of *PHACTR1* significantly activated NF-κB activity compared with control group (**Figure 2G**). After NF-κB activation, the adhesion of THP1 monocytes to ECs is also a vital mechanism of driving endothelial inflammation. We observed that the overexpression of *PHACTR1* could aggravate THP1 cells adhesion to ECs under TNF-α stimulation (**Figure 2H**).

### *PHACTR1* overexpression leads to eNOS downregulation and decreased NO production

Endothelial dysfunction is characterized by reduced bioavailability of NO, which is an early event in the development of atherosclerosis (31). NO is a potent vasodilator and anti-inflammatory signal molecule, which is a gasotransmitter produced by endothelial NO synthases (eNOS) in a stepwise redox reaction from L-arginine (32). NO plays a variety of roles in maintaining vascular homeostasis (33). In contrast, endothelin 1 (EDN1) is the most potent vasoconstrictor which induces the expression of proinflammatory signals and promotes vasoconstriction (34). EDN1 could constrict blood vessels in inflamed areas to contribute to cardiovascular diseases (6).

**Figure 3:**
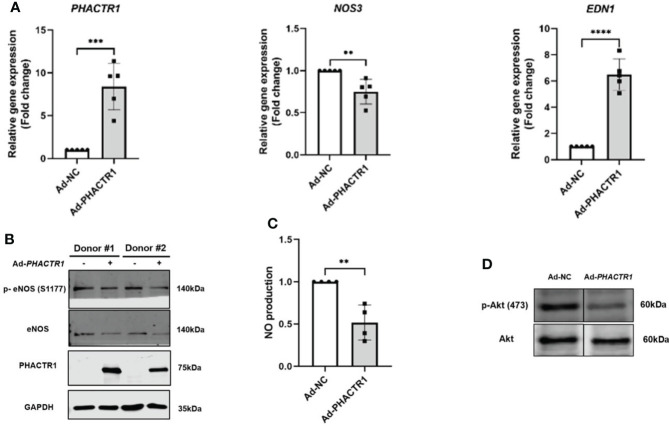
PHACTR1 overexpression leads to eNOS downregulation and decreased NO production. **(A)** PHACTR1 overexpression upregulated EDN1 (also known as ET-1) and downregulated eNOS mRNA expression in HUVECs (n = 5). **(B)** PHACTR1 overexpression can decrease eNOS total protein and eNOS phosphorylation at Ser1177 (n = 6). **(C)** PHACTR1 overexpression can reduce NO production in HUVECs (n = 4). **(D)** PHACTR1 overexpression decreased Akt phosphorylation at Ser473 (n = 3). Lanes presented were spliced from the same blots with uncropped images displayed in online **Supplementary Material**. **P < 0.01, ***P < 0.001, ***P < 0.0001 ****, P < 0.0001.

Taking that into account, we evaluated the effect of PHACTR1 on eNOS expression after treatment with Ad-*PHACTR1* in HUVECs. *PHACTR1* overexpression decreased the level of eNOS gene and protein expression (**Figures 3A, B**). Also, the expression of eNOS phosphorylation at Ser1177 (p-eNOS-S1177) was decreased accordingly (**Figure 3B**). Similarly, the level of EDN1 gene expression was significantly elevated (**Figure 3A**). Next, we assessed the possible effect of *PHACTR1* overexpression on NO production in HUVECs. We observed that NO production was significantly reduced by *PHACTR1* overexpression in HUVECs (**Figure 3C**). To the best of our knowledge, this is the first study to assess the effect of *PHACTR1* on endothelial NO production. It is known that Akt mediates eNOS phosphorylation and increases VEGF secretion, vasodilation, and angiogenesis in the cardiovascular system (35). We observed that the expression of Akt phosphorylation at Ser473 (p-Akt -Ser473) was decreased by *PHACTR1* overexpression, rather than the total Akt protein expression (**Figure 3D**). At this point, PHACTR1 leads to eNOS downregulation and decreases NO production partially through inhibiting Ser473 phosphorylation of Akt protein to reduce eNOS activation in HUVECs. In this manner, PHACTR1 impairs endothelial NO production, which in turn leads to endothelial dysfunction.

### Identification of statin as a negative pharmacological modifier of *PHACTR1* gene expression

**Figure 4:**
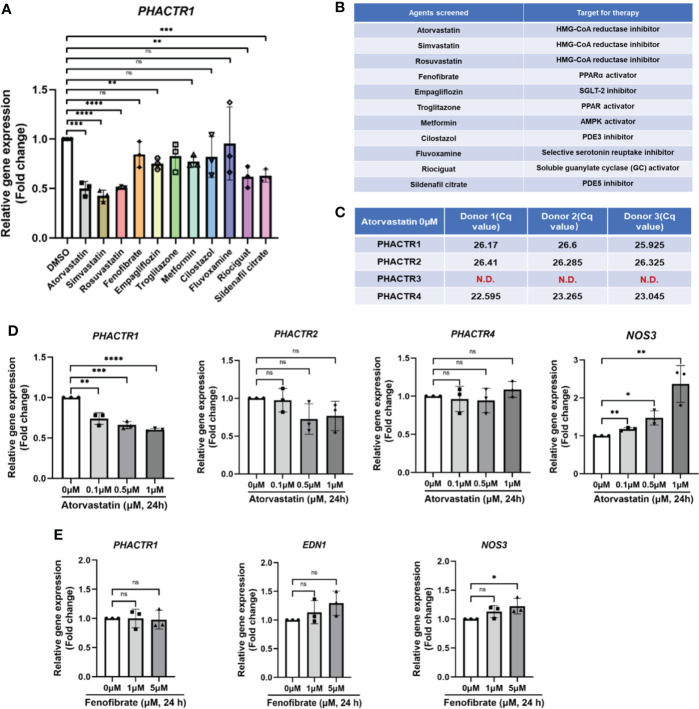
Identification of statin as a negative regulator of PHACTR1 gene expression. **(A)** Effect of selected 11 compounds in in-house library on PHACTR1 gene expression (n = 3). **(B)** Compound list and their molecular targets. **(C)** Gene expression of all PHACTR family members (PHACTR1 to PHACTR4) in HUVECs by real-time PCR (n = 3). **(D)** Effect of atorvastatin on PHACTR1-4 gene expression in HUVECs. NOS3 (eNOS) was used as the positive control (n = 3). **(E)** Effect of Fenofibrate on PHACTR1 gene expression in HUVECs (n = 3). ns, non-significant, N.D., non-detectable, *P < 0.05, **P < 0.01, ***P < 0.001, ***P < 0.0001, ****P < 0.0001.

Since we have confirmed that PHACTR1 could mediate endothelial inflammation and impair endothelial NO production, we next asked whether several clinical agents with known cardiovascular actions could suppress *PHACTR1* gene expression. We performed qPCR validation on 11 selected lipid-lowering, or hypoglycemic or cardiovascular protective drugs. The results showed that Statins, Empagliflozin, Riociguat and Sildenafil citrate had significant inhibitory effects on *PHACTR1* gene expression (**Figures 4A, B**). Considering Atorvastatin has superior effects on lipid-lowering and cardiovascular protection, we focused on evaluating the effect of Atorvastain on *PHACTR1* gene expression (36, 37). Firstly, we observed that only *PHACTR3* was not expressed among four members of the *PHACTR* family in HUVECs (**Figure 4C**). Then, ECs were treated with different doses of Atorvastatin. The results demonstrated that *PHACTR2* and *PHACTR4* had no significant dose-response effects, while only *PHACTR1* expression was decreased by Atorvastatin in a dose-dependent manner (**Figure 4D**). However, another lipid-lowering agent-Fenofibrate with an array of cardiovascular and renal pleiotropic beneficial activities (38), did not have the dose-dependent effects on *PHACTR1* gene expression (**Figure 4E**). Therefore, Statins are likely to be a class of drugs that negatively regulate *PHACTR1* gene expression in human ECs.

### *KLF2* and *KLF4* suppress *PHACTR1* gene expression

**Figure 5:**
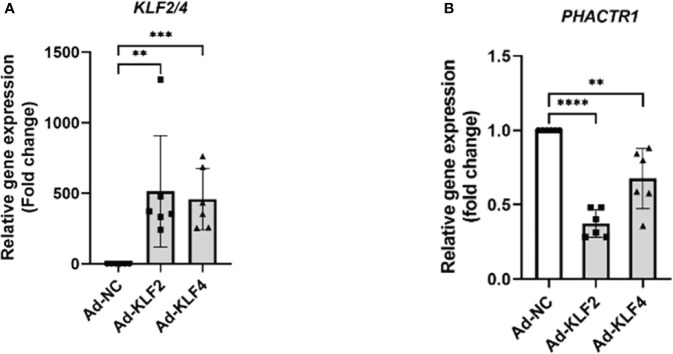
KLF2 and KLF4 suppress PHACTR1 gene expression. **(A)** Validation of KLF2 and KLF4 gene overexpression by adenovirus (n = 6). **(B)** PHACTR1 gene expression was suppressed by Ad-KLF2 and Ad-KLF4 after 24 h of transfection (n = 6). **P < 0.01, ***P < 0.001, ****P < 0.0001.

Since statins are known activators of KLF2 and KLF4, an important class of transcription factors that maintain endothelial homeostasis (39, 40). Though overexpressing *KLF2* and *KLF4*, we found that *PHACTR1* gene expression was significantly downregulated, thus the result further demonstrated that *PHACTR1* could possibly mediated the effects of KLF2 and KLF4 on endothelial homeostasis (**Figures 5A, B**). Considering our RNA-sequencing analysis that PHACTR1 overexpression decreased KLF2 mRNA expression (**Figure 1F**), it is plausible that the effects of eNOS gene expression downregulation by PHACTR1 overexpression may be related to KLF2 downregulation as demonstrated by the fact that activation of KLF2 in ECs induces eNOS gene expression and provides vasodilatory effect (41).

### PHACTR1 interactomics in TNF-α-induced HUVECs

**Figure 6:**
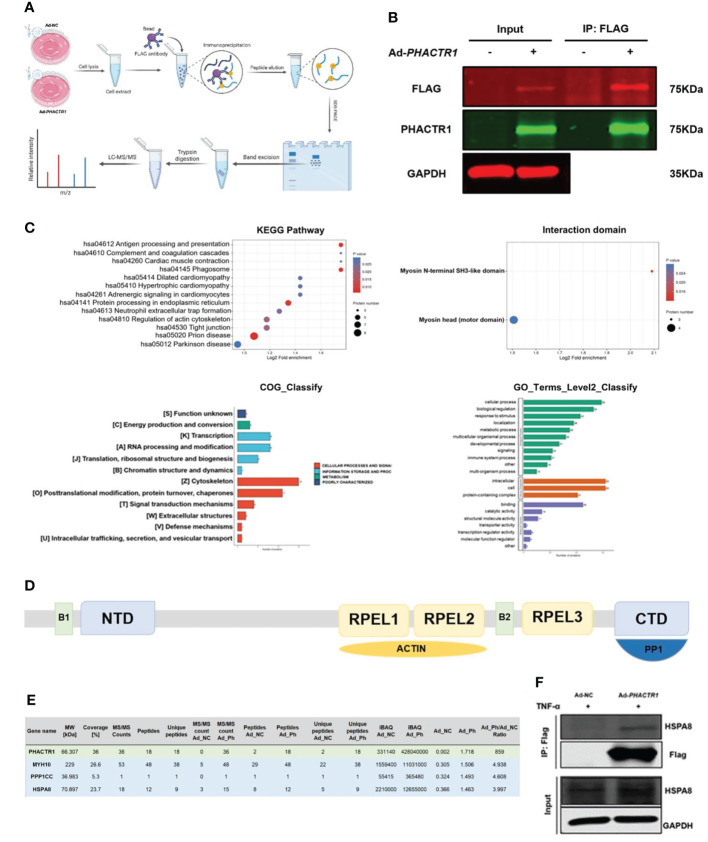
PHACTR1 interactomics in TNF-α-induced HUVECs. **(A)** Scheme of Co-IP study design created with Biorender.com. **(B)** Successful IP of PHACTR1 in TNF-α-treated (10 ng/mL, 6 h) HUVECs revealed by Li-Cor Infrared Imaging. **(C)** GO and KEGG analysis of PHACTR1 interactomics. **(D)** Schematic diagram of PHACTR family protein sequence elements. **(E)** PHACTR1 potential interacting protein were selected by Co-IP. **(F)** Co-IP of PHACTR1 and HSPA8 in HUVECs treated with TNF-α (10 ng/mL, 6 h).

Next, we aim to explore the potential molecular mechanism whereby PHACTR1 triggers endothelial dysfunction. **Figure 6A** is scheme of co-immunoprecipitation (IP) study design. By the successful immunoprecipitation of PHACTR1 in TNF-α-induced HUVECs with *PHACTR1* overexpression (**Figure 6B**), we found that most of the proteins binding to PHACTR1 were proteins with myosin head based on analysis of mass spectrometry (**Figure 6C**). According to the reported binding sites of PHACTR family protein sequence elements, RPEL1 and RPEL2 bind to actin protein, and C-terminal bind to PP1 protein (**Figure 6D**) (42). Three proteins, myosin heavy chain 10 (MYH10), protein phosphatase 1 catalytic subunit gamma (PPP1CC) and heat shock protein family A member 8 (HSPA8), were screened from the table of more than 70 proteins binding with PHACTR1 quantified by mass spectrometry (**Figure 6E**, **Supplementary Table 3**). Through immunoprecipitation-based verification, we observed HSPA8 (HSC70) bind with PHACTR1 (**Figure 6F**). It is well established that HSPA8 is a molecular chaperone that recognizes the non-natural decoupling strands of peptide chains and assists in the correct folding, assembly, transport and degradation of other protein polypeptide chains (43). There is a study demonstrating that HSPA8 and ICAM-1 functioned as damage-induced mediators of γδ T cell activation (44). Moreover, HSPA8 was the most significantly upregulated in the endometrial carcinoma tissues (45). Further studies are warranted to prove whether PHACTR1-HSPA8 interaction is essential for the effects of PHACTR1 on endothelial function, including inflammation and NO production.

## Discussion

ECs act as the gatekeeper of vascular health due to a semipermeable biomechanical barrier between blood flow and vascular wall. Endothelial dysfunction is considered a marker of many different polyvascular diseases in human, including atherosclerosis, hypertension, and diabetes (5).

It was previously reported that *PHACTR1* silencing induced several factors associated with atherosclerotic events, as well as the expression of atherosclerotic biomarkers, and inhibited metabolic response pathways (AMPK/CREB/eNOS) in HUVECs (19). These findings suggest that PHACTR1 might play an anti-inflammatory and anti-atherosclerotic role in ECs (19). However, other evidence suggested that PHACTR1 played a pro-inflammatory and pro-atherogenic role in ECs. To be specific, oxLDL and TNF-α upregulated *PHACTR1* intermediate transcripts (25). Knockdown of *PHACTR1* decreased the expression of inflammatory markers ICAM1, VCAM1 and VE-cadherin induced by oxLDL in human coronary artery ECs. It also alleviates nuclear accumulation of NF-κB/p65 by decreasing its interaction with myocardin-related transcription factor (MRTF-A), thereby inhibiting oxidative stress and inflammation in ECs (20). In spite of above conflicting findings, our results illustrated that PHACTR1 positively regulates TNF-α-induced endothelial inflammation and impairs NO bioavailability by decreasing Akt phosphorylation and eNOS activation. The lack of L-arginine or the essential cofactor tetrahydrobiopterin or excessive oxidative stress, including chronic endothelial inflammation, could lead to eNOS uncoupling (46). In this case, eNOS uncoupling produces superoxide anion and reactive oxygen species (ROS), leading to atherosclerotic plaque formation (6, 47). Based on the above heterogeneity of studies, it may be necessary to study the transcript and genotype of *PHACTR1* firstly and then explore the specific role of PHACTR1 in endothelial inflammation and dysfunction. For example, rs9349379-A/G is located in the non-coding region of PHACTR1 (48). The intermediate transcripts are pro-inflammatory and may differ in their transcriptional regulation levels. Certainly, we cannot totally exclude that the heterogeneity of the results stem from the heterogeneity of ECs from different vascular beds (25, 49).

Considering that the biological function of PHACTR1 itself as a PP1 binding protein, PHACTR1 can directly interact with PP1 and dephosphorylate substrates. Fedoryshchak *et al.* examined the structure of the PHACTR1/PP1 complex using biochemical methods and X-ray crystallography (50). The physical structure showed that the combination of PHACTR1 and PP1 created a new surface pocket. Further experiments showed that the pocket structure allowed the complex to show an order of magnitude enhanced dephosphorylation of its substrate protein compared to PP1 solely (50). However, no more studies have been reported the dephosphorylated role of PHACTR1/PP1 complex in ECs. It is plausible that PHACTR1/PP1 interaction is likely to dephosphorylate Akt at Ser473 or eNOS at Ser1177 to downregulate eNOS activity and decrease NO production. Moreover, HSPA8 (HSC70) belonging to Hsp70 family, acts as a chaperone and is likely to bind PP1 or PHACTR1 to help it assemble correctly for stabilizing its conformation. Moreover, Subramani *et al.* reported that chaperone HSPA8 interacted with cysteine residues of glutathionylated eNOS and shuttle to LAMP2A vesicles to amount chaperone-mediated autophagy in myocardial ischemia-reperfusion injury, leading to irreversible loss of eNOS and NO availability (51). In addition, another GWAS revealed that HSPA8 genetic variant (SNP rs2236659) is associated with coronary heart disease risk in a Chinese population (52). In platelets adhering to collagen, HSPA8 is completely dephosphorylated and dissociates from the Hsp90/PP1α/PP1M complex, suggesting that HSP and PP1 are involved in platelet adhesion (53). It remains to be elucidated whether PHACTR1, PP1 and HSPA8 (HSC70) constitute a trimeric complex to target substrate proteins for dephosphorylation.

Recently, Rubin *et al.* provided an important *in vivo* analysis showing that *Phactr1* knockout separately in ECs or vascular smooth muscle cells do not lead to vascular phenotypes in models of non-atherosclerotic arteriopathies including cervical artery dissection or fibromuscular dysplasia (54). This finding is at variance with intronic variation rs9349379 of *PHACTR1* increases cervical artery dissection and fibromuscular dysplasia risk (55). For noncoding GWAS variants at 6p24 locus, further experiments in atherosclerotic disease models will be required to establish a causal link between *Phactr1* and atherosclerosis. Besides, *Phactr1* has recently been demonstrated to play a key structural role within the vasculature rather than impacting endothelial function (56). To shed light on the role of PHACTR1 in polyvascular diseases, *in vivo* functional assays of *Phactr1* in different vascular disease models will be required using tissue-specific transgenic or deficient mice.

In view of the important role of PHACTR1 in polyvascular diseases, it is vital to study its function and mechanism in the vasculature in future studies (11). Drug discovery with PHACTR1 as therapeutic target is expected and still needs further exploration. From our small-scale drug screening, we found that Statins, Empagliflozin, Riociguat and Sildenafil citrate can decrease expression of *PHACTR1* gene. For genetic variant of rs9349379 (A/G) or mutations at other locus discovered in the future, CRISPR/Cas9 gene editing technology can be used to depict the role of these variants in endothelial function and vascular disorders. In addition, it has been reported that *PHACTR1* gene expression was reduced by 35% by deleting the MEF2 binding site of *PHACTR1* using the CRISPR/Cas9 gene editing technology in ECs carrying this deletion (57). Since KLF2 is transcriptionally regulated by MEF-2, it is also plausible that MEF-2 regulates *PHACTR1* gene expression *via* KLF2.

In conclusion, our study demonstrates the role of PHACTR1 in endothelial dysfunction, in terms of endothelial inflammation and endothelial NO production by activating NF-κB and decreasing Akt/eNOS activity, respectively. A potential limitation of the current study is that we have not validated the assumption of the PHACTR1/PP1α/HSPA8 complex and whether PHACTR1 enhances the binding of HSPA8 to PP1α and orchestrated downstream dephosphorylation events. It also warrants further study whether endothelial cell-derived PHACTR1, unlike that macrophage-derived PHACTR1 (15, 16), can promotes endothelial dysfunction and vascular disease *in vivo*. Addressing these open questions will definitely contribute to our deepened understanding of cell type-specific role of PHACTR1 in endothelial inflammation, innate immunity and associated vascular disorders.

## Data availability statement

The datasets presented in this study can be found in online repositories. The names of the repository/repositories and accession number(s) can be found below: ProteomeXchange Consortium *via* the PRIDE partner repository with the dataset identifier PXD034213 and Gene Expression Omnibus (GEO) repository with the accession number: GSE186761.

## Ethics statement

The studies involving human participants were reviewed and approved by Institutional review board of First Affiliated Hospital of University of Science and Technology of China. The patients/participants provided their written informed consent to participate in this study. The animal study was reviewed and approved by Institutional Animal Care and Use Committee of University of Science and Technology of China (USTC).

## Author contributions

SX and JW conceptualize and supervise whole study. XM, MS, ZZ, QH, ZL and FZ performed experiments and data analysis. LS provide insightful comments on the manuscript. All authors read, revised, and approved the final manuscript.

## Funding

This study was supported by grants from the National Key R&D Program of China (No.2021YFC2500500), the National Natural Science Foundation of China (Grant Nos. 82070464, 81941022, 81530025), and the Strategic Priority Research Program of Chinese Academy of Sciences (Grant No. XDB38010100). This work was also supported by the Program for Innovative Research Team of the First Affiliated Hospital of USTC (CXGG02), Anhui Provincial Key Research and Development Program (Grant No. 202104j07020051), Anhui Province Science Fund for Distinguished Young Scholars (Grant No. 2208085J08), Local Innovative and Research Teams Project of Guangdong Pearl River Talents Program (Grant No. 2017BT01S131).

## Acknowledgments

Scheme of **Figure 6A** was created using Biorender.com.

## Conflict of interest

The authors declare that the research was conducted in the absence of any commercial or financial relationships that could be construed as a potential conflict of interest.

## Publisher’s note

All claims expressed in this article are solely those of the authors and do not necessarily represent those of their affiliated organizations, or those of the publisher, the editors and the reviewers. Any product that may be evaluated in this article, or claim that may be made by its manufacturer, is not guaranteed or endorsed by the publisher.
