# Supplementary material for: PHACTR1, a coronary artery disease risk gene, mediates endothelial dysfunction
Source: Front Immunol. 2022 Aug 25;13:958677. doi: 10.3389/fimmu.2022.958677 (PMC9457086; doi:10.3389/fimmu.2022.958677)

Supplemental Materials

Supplemental Figure 1

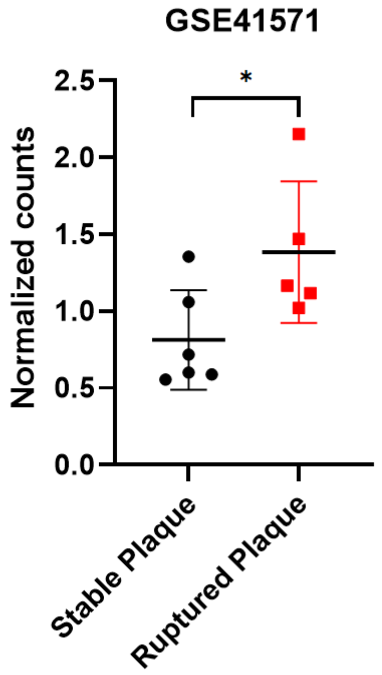

Figure S1. *PHACTR1* gene expression was upregulated in macrophage-rich regions of ruptured human atheromatous plaques (using laser micro-dissection), compared with that of stable plaques by mining GEO datasets (GSE41571) [23].

Supplemental Figure 2

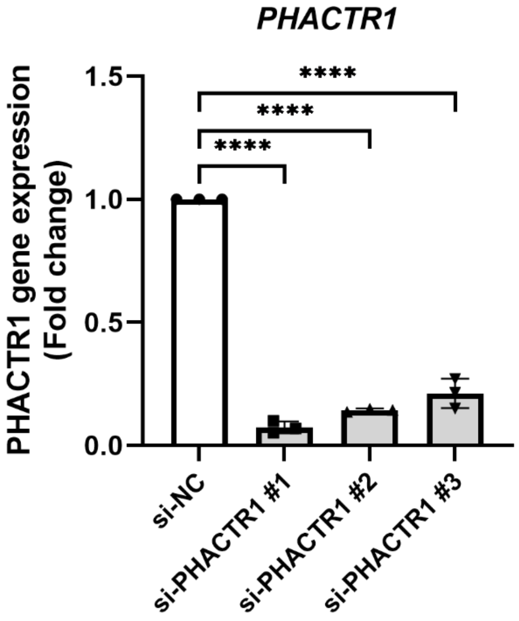

Figure S2. HUVECs were transfected with control siRNA (si-NC) or three independent siRNAs (#1-#3) targeting human PHACTR1 for 48 h before RNA was collected for real-time PCR analysis, n=3. After validation, si-PHACTR1#1 was selected for functional assays in this study. ****P<0.0001.

Supplemental Table 1-Primer sequence

Primers	Sequences
hNOS3-S	CCGGAACAGCACAAGAGTTA
hNOS3-AS	GTCTGTGTTACTGGACTCCTTC
hKLF2-S	GCCGTCCTTCTCCACTTTC
hKLF2-AS	GAAGTCCAGCACGCTGTT
hKLF4-S	GTAGTGCCTGGTCAGTTCATC
hKLF4-AS	GAACCCACACAGGTGAGAAA
hVCAM1-S	GATTGGTGACTCCGTCTCATT
hVCAM1-AS	CCTTCCCATTCAGTGGACTATC
hICAM1-S	CCTCAGCACGTACCTCTATAAC
hICAM1-AS	GGCTTGTGTGTTCGGTTTC
hGAPDH-S	GTCAACGGATTTGGTCGTATTG
hGAPDH-AS	TGTAGTTGAGGTCAATGAAGGG
hPHACTR1-S	GGAACTGGAACAGAGGAACAT
hPHACTR1-AS	TTGACTGAGCTTTCGGGTTAG
hPHACTR2-S	GGAGGAACAGGCAGAAGATAAG
hPHACTR2-AS	TAGGCTTAGGAGGAGCAGAA
hPHACTR3-S	GCGCTGAACGACTCCATTAT
hPHACTR3-AS	CCGGTCTTCTAGTTCCTGTTTG
hPHACTR4-S	CATTCCATCAACCTCAGTACCC
hPHACTR4-AS	GTAGTAGTTCCGAGCCCATAGA
hET1-S	CAAGCAGGAAAAGAACTC
hET1-AS	CTGGTTTGTCTTAGGTGTTC
hSELE-S	GTGTATGTCCTCTGGAGAATGG
hSELE-AS	GAACCCATTGGCTGGATTTG
mGAPDH-S	AGGTCGGTGTGAACGGATTTG
mGAPDH-AS	TGTAGACCATGTAGTTGAGGTCA
mPHACTR1-S	GAGGAGAAGCGGGAAATCAA
mPHACTR1-AS	GTAGTCACTGAAGCGGATAAGG

Supplemental Table 2-Antibodies and sources

Antibody	Catlog# and Vendor
PHACTR1	#23446-1-AP, Proteintech; #ab229120, Abcam
VCAM1	#383318, Zenbio
ICAM1	#200350-F12, Zenbio
GAPDH	#60004-1-Ig, Proteintech
eNOS-pS1177	#612393, BD Biosciences
eNOS	#610297, BD Bioscience
Akt	#342529, Zenbio
Akt-pS473	#381555, Zenbio
FLAG	#250111, Zenbio
HSPA8	#ET1602-33, Huabio

Supplemental Table 3-PHACTR1 interactomics, please see separate excel spreadsheet in online supplemental material.

Uncropped full gels scan

Figure 2C

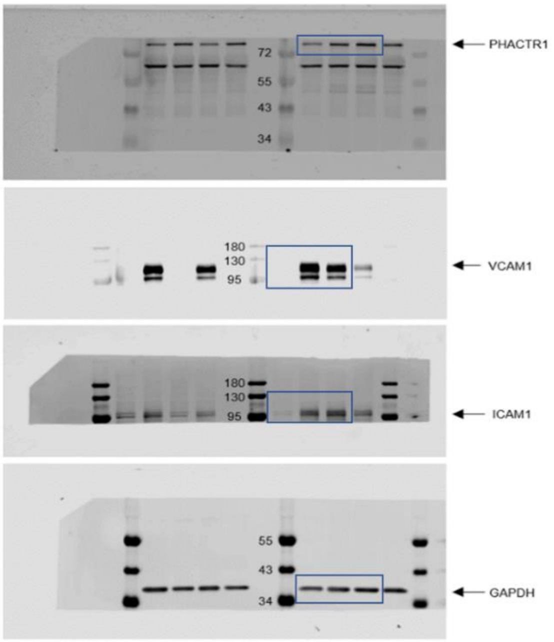

Figure 2E

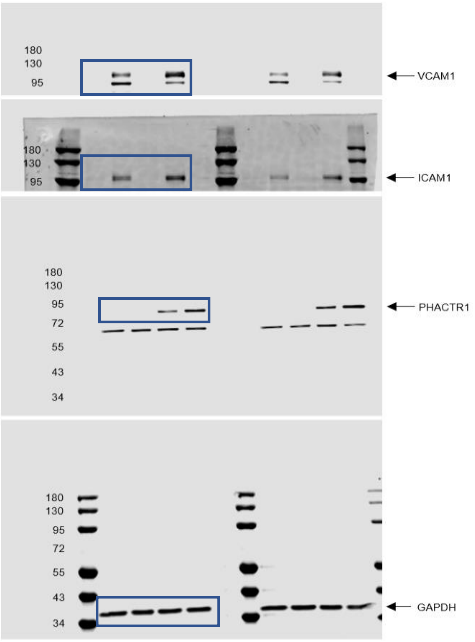

Figure 2F

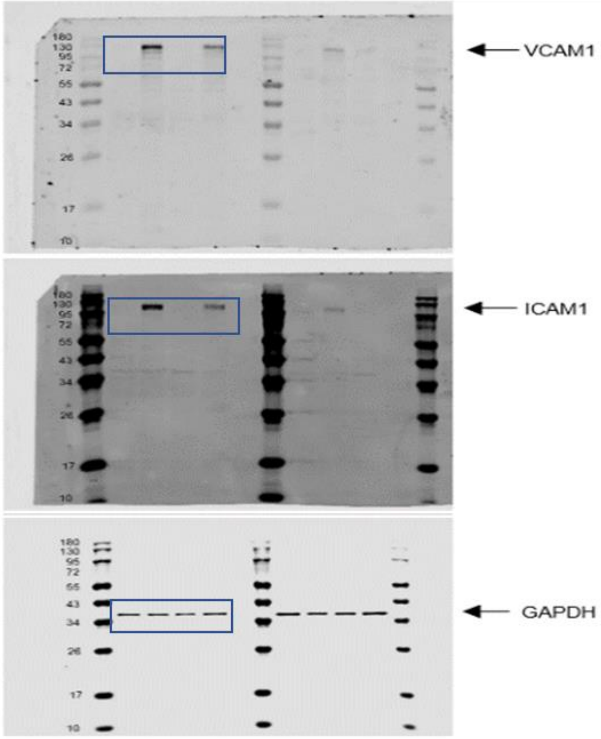

Figure 3B

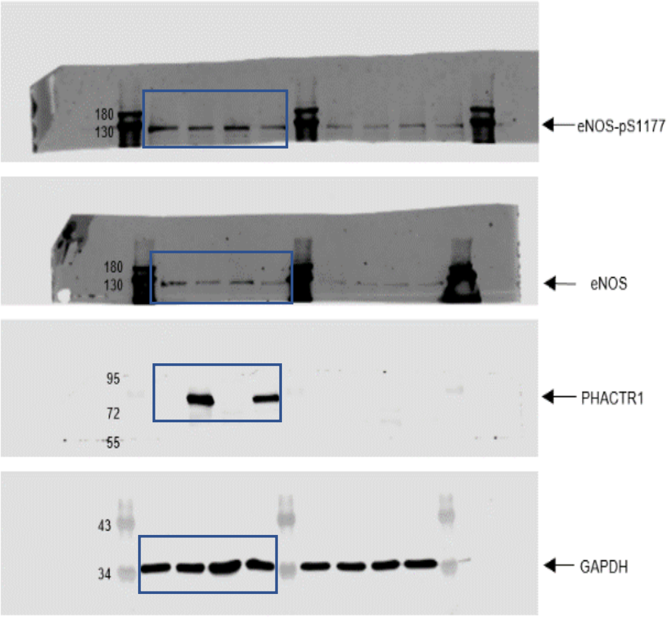

Figure 3D

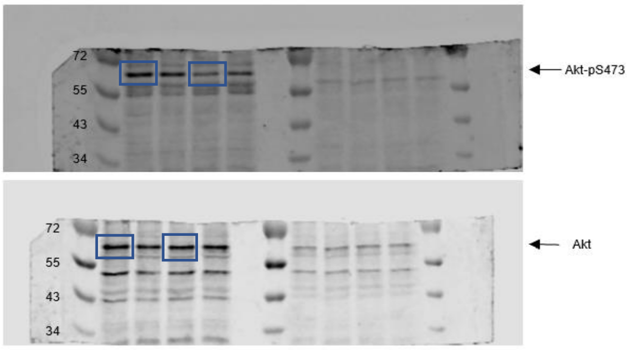

Figure 6B

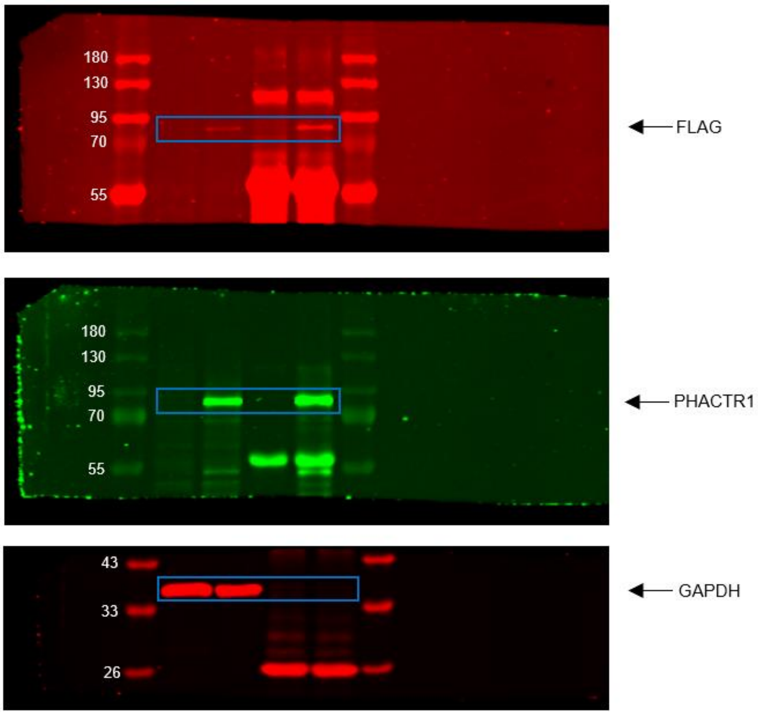

Figure 6F

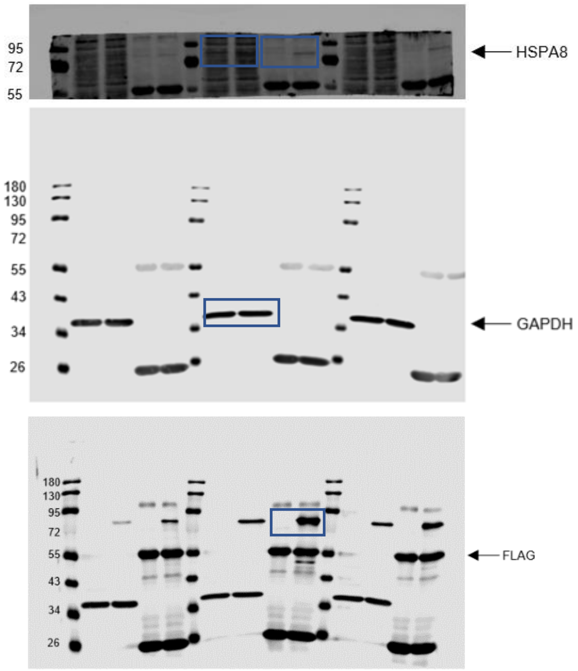

Supplement: Supplementary file 1 [file Presentation_1.pdf]
